# Clinical outcomes in patients with diabetes and stress hyperglycemia that developed SARS-CoV-2 infection

**DOI:** 10.7705/biomedica.7095

**Published:** 2024-05-31

**Authors:** Karen M. Fériz-Bonelo, María B. Iriarte-Durán, Oscar Giraldo, Luis G. Parra-Lara, Veline Martínez, María A. Urbano, Guillermo Guzmán

**Affiliations:** 1 Servicio de Endocrinología, Departamento de Medicina Interna, Fundación Valle del Lili, Cali, Colombia Fundación Valle del Lili Fundación Valle del Lili Cali Colombia; 2 Facultad de Ciencias de la Salud, Universidad Icesi, Cali, Colombia Universidad Icesi Universidad Icesi Cali Colombia; 3 Centro de Investigaciones Clínicas, Fundación Valle del Lili, Cali, Colombia Fundación Valle del Lili Fundación Valle del Lili Cali Colombia; 4 Departamento de Medicina Interna, Fundación Valle del Lili, Cali, Colombia Fundación Valle del Lili Fundación Valle del Lili Cali Colombia

**Keywords:** SARS-CoV-2, COVID-19, diabetes mellitus, hyperglycemia, intensive care units, mortality., SARS-CoV-2, COVID-19, diabetes mellitus, hiperglucemia, unidades de cuidados intensivos, mortalidad.

## Abstract

**Introduction.:**

Diabetes and stress hyperglycemia have been related with poorer clinical outcomes in patients infected by SARS-CoV-2 and at risk for severe disease.

**Objective.:**

To evaluate clinical outcomes in three groups of patients (with diabetes, without diabetes and with stress hyperglycemia) with SARS-CoV-2 infection.

**Materials and methods.:**

A retrospective cohort study was conducted in Cali (Colombia). We included patients 18 years old or older with a diagnosis of SARS-CoV-2 infection, managed in the emergency room, hospitalization, or intensive care unit between March 2020 and December 2021. Immunocompromised patients and pregnant women were excluded. Patients were classified into three groups: without diabetes, with diabetes, and with stress hyperglycemia. A comparison between the groups was performed.

**Results.:**

A total of 945 patients were included (59.6% without diabetes, 27% with diabetes, and 13.4% with stress hyperglycemia). Fifty-five-point three percent required intensive care unit management, with a higher need in patients with stress hyperglycemia (89.8%) and diabetes (67.1%), with no difference between these groups (p = 0.249). We identified a higher probability of death in the group with stress hyperglycemia versus the one without diabetes (adjusted OR = 8.12; 95% CI: 5.12-12.88; p <0.01). Frequency of acute respiratory distress syndrome, need for invasive mechanical ventilation, use of vasopressors and inotropes, need for *de novo* renal replacement therapy, and mortality was higher in patients with metabolic alterations (diabetes and stress hyperglycemia).

**Conclusions.:**

Diabetes and stress hyperglycemia were associated with worse clinical outcomes and mortality in patients with COVID-19. These patients should be identified early and considered them high risk at the COVID-19 diagnosis to mitigate adverse outcomes.

## Resumen

**Introducción.:**

La diabetes y la hiperglucemia de estrés se han relacionado con peores desenlaces clínicos en pacientes infectados por SARS-CoV-2 y con riesgo de enfermedad grave.

**Objetivo.:**

Evaluar los resultados clínicos en tres grupos de pacientes (con diabetes, sin diabetes o con hiperglucemia de estrés) con infección por SARS-CoV-2.

**Materiales y métodos.:**

Se llevó a cabo un estudio retrospectivo de cohorte en Cali (Colombia). Se incluyeron pacientes de 18 años o más, con diagnóstico de infección por SARS-CoV-2 atendidos en urgencias, hospitalización o unidad de cuidados intensivos entre marzo de 2020 y diciembre de 2021. Se excluyeron los pacientes inmunocomprometidos y las mujeres embarazadas. Los pacientes fueron clasificados en tres grupos: sin diabetes, con diabetes y con hiperglucemia de estrés. Se realizó una comparación entre los grupos.

**Resultados.:**

Se incluyeron 945 pacientes (59,6 % sin diabetes, 27 % con diabetes y 13,4 % con hiperglucemia de estrés). El 55,3 % requirió manejo en la unidad de cuidados intensivos, con mayor necesidad por parte de los pacientes con hiperglucemia de estrés (89,8 %) y diabetes (67,1%), sin diferencia entre estos grupos (p = 0,249). Se observó una mayor probabilidad de muerte en el grupo con hiperglucemia de estrés versus sin diabetes (OR ajustado = 8,12; IC_95%_: 5,12-12,88; p <0,01). La frecuencia de síndrome de distrés respiratorio agudo, necesidad de ventilación mecánica invasiva, uso de vasopresores e inotrópicos, necesidad de terapia de reemplazo renal *de novo* y mortalidad fue mayor en pacientes con alteraciones metabólicas (diabetes e hiperglucemia de estrés).

**Conclusiones.:**

La diabetes y la hiperglucemia de estrés se asociaron a peores resultados clínicos y mortalidad en pacientes con COVID-19. Estos pacientes deben ser identificados tempranamente y considerados de alto riesgo al momento del diagnóstico de COVID-19 para mitigar los desenlaces adversos.

In December 2019, the world saw how SARS-Cov-2 infection started taking thousands of lives ^1^ being the COVID-19 epidemic declared a public health emergency by the World Health Organization (WHO) on January 30^th^, 2020 and characterized as a pandemic on March 11^th^, 2020 ^2^. In Colombia, according to the *Instituto Nacional de Salud*, the number of confirmed cases was 6,305,562 with a total of 141,746 deceased patients ^3^.

The mechanisms of glycemic disturbances in COVID-19 include several complex and interrelated etiologies, including impairments in glucose disposal and insulin secretion, stress hyperglycemia, preadmission diabetes, and steroid-induced diabetes. Additionally, factors that have been identified, such as preexisting diabetes, poor glycemic control (age, sex, comorbidities, obesity, inflammation, pro-coagulative state), COVID-19 severity (SARS-CoV-2 (B-cell tropism, cytokine storm, stress) that contribute to new-onset diabetes show a bidirectional relationship between type 2 diabetes, hyperglycemia, and COVID-19 ^4^. That is why diabetes is a risk factor for developing severe COVID-19 with a higher risk of related adverse outcomes ^5^^-^^8^.

Severe hyperglycemia is common in critically ill patients and is often a marker of disease severity ^9^. Stress hyperglycemia negatively affects the outcomes of patients with and without diabetes hospitalized due to infections. Evidence suggests that stress hyperglycemia alters the immune response against infection, increases the release of pro-inflammatory chemokines, generates abnormalities in the coagulation system, increases oxidative stress, induces greater bronchial hyperreactivity, and promotes airway fibrosis ^10^.

As for the greater risk in patients with metabolic alterations of glucose, such as diabetes and stress hyperglycemia in the current SARS-CoV-2 pandemic scenario, there are further studies needed in different population groups that allow the establishment of expected clinical outcomes for each one. This study aimed to evaluate the clinical outcomes in patients with diabetes and stress hyperglycemia who developed SARS-CoV-2 infection.

## Materials and methods

### Design and setting

A retrospective cohort study was conducted at the Fundación *Valle del Lili*, in Cali (Colombia), a non-profit university hospital serving as a reference center for all the Colombian southwest, affiliated with the *Facultad de Ciencias de la Salud from the Universidad ICESI*. In Colombia, the prevalence of diabetes in 2021 was around 10%, according to the International Diabetes Federation ^11^. The high-cost account reported the incidence of diabetes in men as 2.98 and 3.77 in women per 100,000 inhabitants. The highest proportion of newer cases occurs between 55 and 69 years of age, accounting for 43.77% of incidence ^12^.

In the country, most COVID-19 cases occurred in the age group that comprises between 30 and 39 years: 52.52% corresponded to women, 97.01% were mild cases, the death rate was 2.5 per 100 cases, and the three main comorbidities were hypertension (6,416), diabetes (3,901), and kidney disease (2,226 cases) ^13^.

### Ethics statement

The *Comité de Ética en Investigación Biomédica at Fundación Valle del Lili* approved this study (IRB/EC 1566), and it was conducted after the Declaration of Helsinki and Resolution 8430/1993 from the Colombian *Ministerio de Salud y Protección Social*. There was no process of written consent, because data was gathered through clinical records and databases from the clinical and microbiology laboratories.

### Patients and data

The selected population were patients treated between March 2020 and December 2021 for COVID-19. Patients 18 years old or more, from both sexes, admitted to the hospital and managed either in the emergency room, hospitalization or in the intensive care unit were eligible.

### SARS-CoV-2 infection cases

SARS-CoV-2 cases were patients with clinical or epidemiological criteria and a viral antigen detection test, or presence of SARS-CoV-2 antibodies, or patients with a positive viral real-time RT-PCR test assay regardless of clinical or epidemiological criteria (according with the WHO definitions). The clinical criteria were acute onset of fever and cough (influenza-like illness) or acute onset of three or more of any signs or symptoms (fever, cough, weakness/ fatigue, headache, myalgia, sore throat, coryza, dyspnea, nausea, diarrhea or anorexia). The epidemiological criteria were contact of a probable, confirmed or linked case to a COVID-19 cluster.

SARS-CoV-2 infections were diagnosed with nasopharyngeal swabs using the CDC 2019-nCoV real-time RT-PCR diagnostic panel protocol (CDC, Atlanta, Georgia, USA), Viasure® SARS-CoV-2 real-time PCR detection kit (Certest Biotec S.L., Zaragoza, Spain), Allplex™ 2019-nCoV assay (Seegene Inc, Seoul, South Korea), orAccuPower® SARS-CoV-2 multiplex real-time RT-PCR Kit (Bioneer Corporation, Daedeok-gu, South Korea. Measurement of IgG and IgM antibodies against SARS CoV-2 was through a chemiluminescence assay (Abbott Architect Assays, Chicago, Illinois). All diagnosis tests were performed in the hospital, and cases were obtained from the clinical records and laboratory databases.

### Exclusion criteria

Immunocompromised and pregnant patients were excluded.

### Cases classification

Patients included were classified in three groups: without diabetes, with diabetes (known diagnosis, or HbA1c > 6.5%) and with stress hyperglycemia (defined as blood glucose levels > 180 mg/dl and HbA1c <6.5%, or blood glucose levels > 180 mg/dl, without HbA1c measurement during the hospitalization). This cut-off to define stress hyperglycemia is based on the criteria of some scientific associations like the Endocrine Society ^14^; it is also the maximum upper limit for the initiation of insulin therapy in the hospital setting, and some studies showed there are worse clinical outcomes associated with this level of blood glucose ^15^^,^^16^.

### Variables and outcomes

Demographic, clinical, laboratory tests, treatment (need of insulin, required insulin dose, glycemic control during hospitalization and development of diabetic ketoacidosis) and complications variables were collected retrospectively from the clinical records of all patients. Old age was defined as higher than or equal to 65 years old; cardiovascular event as the group of coronary disease, heart failure and arrythmias; chronic kidney disease as a glomerular filtration rate < 60 mL/min/1.73 m^2^ calculated by the CKD-EPI equation ^17^; hypertension as a patient with a known diagnosis following the criteria given by the Eight Joint National Committee ^18^ or the use of antihypertensive medication. Body mass index (BMI) was determined by weight and height at hospital admission (kg/m^2^).

The clinical outcomes evaluated during the follow-up while the patient was hospitalized were in-hospital stay (intensive care unit and general hospitalization), sequential organ failure assessment (SOFA) score, acute respiratory distress syndrome, need for invasive mechanical ventilation, use of vasopressor and/or inotrope support, *de novo* renal replacement therapy and death. The information related to these outcomes was collected in a database retrospectively after reviewing the medical records.

### Statistical analysis

We performed a descriptive analysis of the data. Data distribution was evaluated with the Shapiro-Wilk test. Numerical variables comparison between groups was performed with the Mann-Whitney’s U test or t of Student regarding the data distribution; the chi squared test was used for categorical variables.

Odds ratios (OR) were calculated with corresponding 95% confidence intervals (CI) through logistic regression for qualitative variables to measure the association. For outcomes involving quantitative variables, (B-coefficients were obtained using linear regression. Graphic representations of serum glucose levels were also provided for each group. Statistically significant differences were considered if the p value was less than 0.05. Performed analyses were further refined by adjusting for potential confounding factors. Specifically, the models were adjusted for heart disease, chronic kidney disease, hypertension, and angiotensin II receptor blocker use.

Study data were collected and managed using REDCap electronic data capture tools hosted at *Fundación Valle del Lili*^19^^,^^20^. All analyses were performed using Stata™, version 14.0 (StataCorp LP, College Station, TX).

## Results

**Figure 1.**
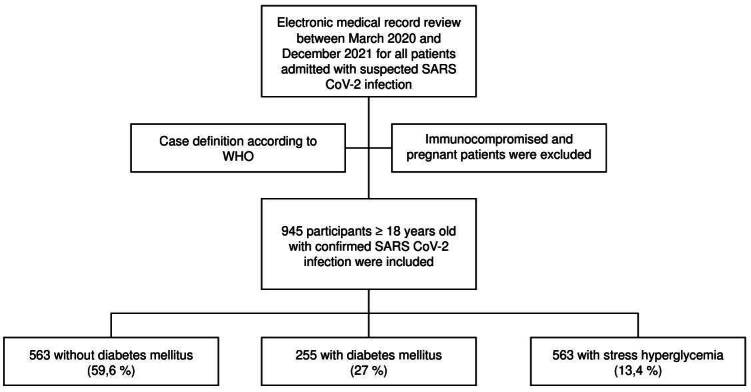
Patient selection flow chart

We included a total of 945 patients with confirmed COVID-19 diagnosis: 563 did not have diabetes (59.6%), 255 had diabetes (27%), and 127 presented stress hyperglycemia (13.4%). The patient selection flow chart is shown in figure 1.

### Population characteristics

Table 1 presents the demographic and clinical characteristics of patients at hospital admission. Ages ranged between 18 and 99 years; the youngest population belonged to the group without diabetes (p < 0.001). Most were men (63.1%), but there was no significant difference regarding sex or BMI in the different groups (median = 27.1 kg/m^2^; IQR: 24.4-30.5 kg/m^2^).

**Table 1:** Demographic and clinical characteristics of patients included in the study (N = 945).

Characteristics		Total (N = 945)		Without diabetes (n = 563		Diabetes n = 255		Stress hyperglycemia n = 127		p-value
Demographic										
	Age (years),									
	median (IQR)	61	(50-72)	57	(45-68)	66	(57-73)	67	(56 - 76)	< 0,001
	Male sex, n (%)	596	(63,1)	347	(61.6)	166	(65.1)	83	(65.4)	0.540
Clinical										
	BMI (kg/m2), median (IQR)	27.1	(24.4-30.5)	27.1	(24.2-30.1)	27.4	(24.8-31.3)	26.8	(24.2-30.1)	0.184
Comorbidities, n (%)										
	Hypertension	427	(45.2)	205	(36.4)	163	(63.9)	59	(46.5)	< 0.001
	Chronic kidney disease	99	(10.5)	41	(7.3)	44	(17.3)	14	(11.0)	< 0.001
	Neoplasms	97	(10.3)	57	(10.1)	25	(9.8)	15	(11.8)	0.819
	Heart disease	96	(10.2)	41	(7.3)	40	(15.7)	15	(11.8)	0.001
	Coronary artery disease	56	(58.3)	20	(48.8)	28	(70.0)	8	(53.5)	0.140
	Cerebrovascular disease	34	(3.6)	20	(3.6)	11	(4.3)	3	(2.4)	0.614
	Chronic obstructive lung disease	32	(3.4)	17	(3.0)	10	(3.9)	5	(3.9)	0.863
	Chronic heart failure	26	(27.1)	10	(24.4)	11	(27.5)	5	(33.3)	0.705
	Arrhythmias	25	(2.6)	8	(1.4)	12	(4.7)	5	(3.9)	0.372
	Pulmonary hypertension	6	(0.6)	5	(0.8)	1	(0.4)	-		0.158
	Smoking, n (%)	71	(7.5)	38	(6.7)	21	(8.2)	12	(9.5)	0.508
Drugs, n (%)										
	ACEI	38	(4)	19	(3.4)	14	(5.5)	5	(3.9)	0.361
	ARB	300	(31.7)	136	(24.2)	122	(47.8)	42	(33.1)	< 0.001
Laboratory										
	Glycated hemoglobin (%), median (IQR)	7.2	(6.5-8.4)	NA		7.3	(6.7-8.7)	6.2	(5.7-6.3)	< 0.001
	Thrombocytopenia (< 150.000/µl) n (%)	41	(4.3)	27	(4.8)	4	(1-6)	10	(7.9)	0.012
	Neutrophil/lymphocyte rate, median (IQR)	6.3	(3.4-11.0)	5.5	(3.0-10.4)	6.7	(3.9-11.1)	8.9	(5.1-14.7)	< 0.001
	C-reactive protein (mg/dl), median (IQR)	11.1	(5.4-21.2)	8.9	(3.9-18.8)	14.1	(6.9-23.8)	15.6	(9.5-25.7)	< 0.001
	Erythrocyte sedimentation rate (mg/dl), median (IQR)	28	(13-40)	22	(13-46)	37	(30-43)	24.5	(12-30)	0.238
	Interleukin 6, median (IQR)	31.1	(9.9-92.9)	28.3	(6.6-91.8)	32.4	(10.2-101)	31.1	(13.4-86.8)	0.830
	D-dimer (µg/ml), median (IQR)	1	(0.6-1.8)	0.9	(0.5-1.5)	1.1	(0.6-2.0)	1.4	(0.9-5.5)	< 0.001
	Ultra-high sensitivity troponin-I (ng/l), median (IQR)	7.7	(3.4-25.9)	5.35	(2.6-16.9)	10.6	(4.4-44.6)	17.9 (	7.9-85.4)	< 0.001

ACEI: angiotensin converting enzyme inhibitor; ARB: angiotensin II receptor blocker; IQR: Interquartile range

Cardiovascular comorbidities were present in 10.2% of patients, being more frequent in patients with diabetes than in the other groups (15.7%; p <0.001). Hypertension was higher in the diabetes group (63.9%; p < 0.001), like chronic kidney disease (17.3%; p < 0.001). There were no differences with smoking, cerebrovascular events, chronic obstructive pulmonary disease (COPD), and neoplasms among groups.

Patients with diabetes and stress hyperglycemia presented a higher increase in the neutrophil/lymphocyte ratio, serum concentration of C-reactive protein, D-dimer, and ultra-high sensitivity cardiac troponin-l compared to the group without diabetes.

When evaluating the population with diabetes (n = 225), we found that metformin was the most used medicine for outpatient management (43% of the cases). Twenty-five-point-nine percent of the patients used insulin at the admission (median insulin dose was 34 IU/day; IQR: 20-50 IU/day), 9.8% received DPP-4 inhibitors, 4.7% SGLT2 inhibitors, 2.7% sulfonylureas, and 1.9% GLP-1 receptor agonist. The median HbA1c was 7.2% (IQR: 6.5-8.42).

### Clinical outcomes during hospitalization

Median in-hospital stay was 11 days (IQR: 5-23 days) for patients with diabetes and 17 days for stress hyperglycemia (IQR: 10-29 days) with a statistically significant difference (p < 0.001); 55.3% of the population required intensive care unit management. The need for intensive care unit transfer was higher in patients with stress hyperglycemia (89.8%) and diabetes (67.1%) than in the group without diabetes (42.3%) as well as intensive care unit stay (12 and 11 days versus 6 days, respectively; p < 0.001) (table 2).

**Table 2.** Level of healthcare attention and stay-in times of the included patients

Characteristics	Total (N = 945)	Without diabetes (n = 536)	Diabetes (n = 255)	Stress hyperglycemia (n = 127)	p value
Inpatient hospital stay (days), median (IQR)	8 (4-18)	6 (3-12)	11 (5-23)	17(10-29)	< 0.001
Transfer to general hospitalization rooms, n (%)	422 (44.7)	325 (57.7)	84 (32.9)	13(10.2)	< 0.001
Stay-in time in general hospitalization rooms (days), median (IQR)	4 (2-7)	4 (2-7)	4 (2-7)	7 (4-11)	0.171
ICU transfer, n (%)	523 (55.3)	238 (42.3)	171 (67.1)	114(89.8)	-
ICU stay-in time (days), median (IQR)	8 (4-16)	6 (3-11)	11 (5-18)	12 (7-22)	< 0.001
Steroid use, n (%)	757 (80.1)	415(73.7)	221 (86.7)	121 (95.3)	< 0.001

IQR: interquartile range. ICU: intensive care unit

Patients with diabetes and stress hyperglycemia had higher chances of acute respiratory distress syndrome, invasive mechanical ventilation need, vasopressor and inotrope support, and *de novo* renal replacement therapy requirement compared to normoglycemic patients (table 3). We found a higher likelihood of death in patients with the previously mentioned abnormalities, differences that kept on showing in the logistic regression model.

**Table 3:** Clinical outcomes of patients with glucose alterations (diabetes and stress hyperglycemia) compared to normoglycemic patients during inpatient hospital stay.

Clinical outcomes		Diabetes OR (95% CI)	Stress hyperglycemia OR (95% CI)
Logistic regression model			
	ARDS	3.35 (2.44-4.60)	7.93 (4.74-13.29)
	Invasive mechanical ventilation	4.20 (3.02-5.84)	16.23 (10.16-25.94)
	Vasopressor requirement	4.53 (3.17-6.46)	10.98 (7.10-16.98)
	Inotrope requirement	5.14(2.86-9.22)	8.96 (4.79-16.76)
	De novo renal replacement therapy requirement	5.38 (3.42-8.46)	4.44 (2.567.68)
	ICU requirement	2.78 (2.04-3.79)	11.97 (6.59-21.77)
	Mortality	3.16 (2.08-4.81)	8.12 (5.12-12.88)
		Diabetes β coefficient (95% CI)	Stress hyperglycemia β coefficient (95% CI)
Linear regression model			
	SOFA score	1.63 (1.10-2.17)	2.30 (1.67-2.93)
	Inpatient stay in general hospitalization rooms	6.62 (3.86-9.37)	13.48 (9.90-17.06)
	ICU stay-in time	5.78 (3.11-8.45)	8.16(5.17-11.16)

ARDS: acute respiratory distress syndrome; ICU: intensive care unit; SOFA: Sequential Organ Failure Assessment.

### Clinical outcomes in intensive care unit

Considering the sample size for each group, adjustments were made solely for heart disease, chronic kidney disease, hypertension, and angiotensin II receptor blocker use; the choice of these specific variables aimed to balance the need for adjustment with maintaining parsimony.

**Figure 2.**
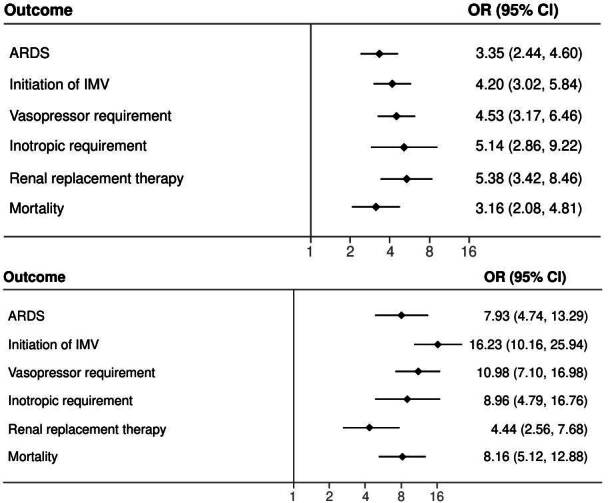
Clinical outcomes of patients treated in the intensive care unit (n = 523). ARDS: acute respiratory distress syndrome; IMV: invasive mechanical ventilation

**Figure 3.**
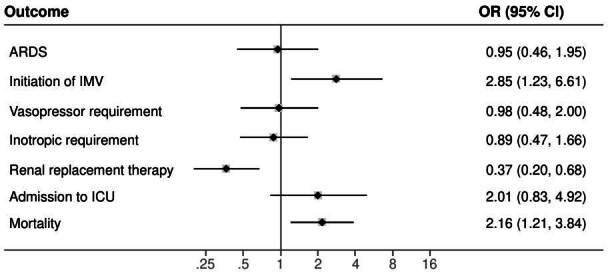
Logistic regression model between diabetes and stress hyperglycemia for clinical outcomes ARDS: acuterespiratory distress syndrome; IMV: invasive mechanical ventilation; ICU: intensive care unit Note: SOFA score and inpatient stay in intensive care unit variables were not included.

Figure 2 presents the clinical outcomes of 523 patients that required intensive care unit management. Patients with diabetes had higher probabilities of developing acute respiratory distress syndrome (OR =3.35; 95% CI: 2.44-4.60), invasive mechanical ventilation requirement (OR = 4.20; 95% CI: 3.02-5.84), vasopressor (OR = 4.5; 95% CI: 3.17- 6.46) and inotrope support need (OR = 5.14; 95% CI: 2.86-9.22), and renal replacement therapy (OR = 5.38; 95% CI: 3.42-8.46) than those with normoglycemia. Patients with stress hyperglycemia had higher probabilities of developing acute respiratory distress syndrome (OR = 7.93; 95% CI: 4.74-13.29), invasive mechanical ventilation requirement (OR = 16.23; 95% CI: 10.16-25.94), vasopressor (OR = 10.98; 95% CI: 7.10- 16.98) and inotrope support need (OR = 8.96; 95% CI: 4.79-16.76), and renal replacement therapy (OR = 4.44; 95% CI: 2.56-7.68) than those with normoglycemia. Differences that kept true after adjusting for the logistic regression model (figure 3). We found that the presence of diabetes (OR = 3.16; 95% CI: 2.08-4.81) and stress hyperglycemia (OR = 8.16; 95% CI: 5.12-12.88) significantly increased the risk of death when compared with those with normoglycemia.

### Body mass index, age, and sex effect

When adjusting the effect of BMI on mortality for those with diabetes versus those without diabetes, we found that the presence of diabetes increased the risk of death independent of BMI. In patients with obesity, the absence of diabetes behaved as a protective factor (OR = 0.40; 95% CI: 0.16-0.97; p = 0.042).

The analysis reported that the risk of death is directly related to age. When suffering from diabetes, mortality increases independent of age, observing a trend worse in those with diabetes and old age (OR = 42.85; 95% CI: 10.18-180.42; p <0.001). In patients from 18-49 years of age, the risk was higher (OR = 12.07; 95% CI: 1.91-76.23; p = 0.008) than in the 50-64 years old group (OR = 8.74; 95% CI: 1.81-42.08; p = 0.007). When adjusted to diabetes, the sex category compelled a significantly higher risk of death in men (OR = 4.54; 95% CI: 2.42-8.53; p <0.001). Nonetheless, both sexes had a higher chance of death when diabetes was present independent of sex.

We discovered that patients with stress hyperglycemia have a higher risk of dying independent of BMI versus those with diabetes, and in patients with obesity, the lack of stress hyperglycemia behaved as a protective factor (OR = 0.39; 95% CI: 0.16-0.97; p = 0.042). In relation to age and sex, the probability of death increases with the presence of stress hyperglycemia, being highest in those older than 65 years; as it happened in the diabetes group, patients with stress hyperglycemia between 18 and 49 years had higher chances of death (OR = 59; 95% CI: 11.26-309.10; p< 0.001) than those between 50-64 years (OR = 28.55; 95% CI: 5.97- 136.59; p <0.001)

Our analysis of mortality revealed that diabetes is an independent risk factor for increased mortality (OR = 2.55; 95% CI: 1.60-4.08; p < 0.001). Moreover, it increased when adjusted to concomitant heart disease if both conditions were present (OR = 8.41; 95% CI: 4.17-16.97; p <0.001). The trend remains when adjusting for chronic kidney disease (OR =7.54; 95% CI: 3.79-14.99; p <0.001) and hypertension (OR = 4.03; 95% CI: 2.33-6.94; p <0.001). With stress hyperglycemia the same findings were obtained, having a higher probability of dying in patients with stress hyperglycemia than those without it, worsening if two pathologies were present (heart disease: OR = 17.07; 95% CI: 5.80-50.27; p < 0.001; chronic kidney disease: OR = 8.98; 95% CI: 2.97-27.16; p < 0.001; hypertension: OR = 9.73; 95% CI: 5.05-18.78; p <0.001).

### Glycemic control

The HbA1c value was obtained before inpatient admission in 149 patients. Median HbA1c for patients with diabetes mellitus was 7.3% (IQR: 6.7-8.7); 11.5% (13/113) of the patients had a value inferior to 6.5%.

In-hospital glycemic control was studied in 374 patients: 86.6% were out of treatment goals. In these patients, there was a higher frequency of acute respiratory distress syndrome, invasive mechanical ventilation, vasopressor support requirement, inpatient hospitalization time, and intensive care unit transfer need (table 4).

**Table 4:** Clinical outcomes according to glycemic control

Characteristics	Glycemic control during hospitalization		p value
	Out of goals (n = 324)	Within goals‡ (n = 50)	
SOFA score, median (IQR)*	4 (3-7)	4 (2-5)	0.081
ARDS, n (%)	260 (80.2)	27 (54.0)	< 0.001
IMV requirement, n (%)	196 (60.5)	21 (42.0)	0.014
Vasopressor requirement, n (%)	-	17 (34.0)	0.047
Inotrope requirement, n (%)	57 (17.6)	9 (18.0)	0.944
De novo renal replacement therapy, n (%)	81 (25.0)	11 (23.4)	0.761
Diabetic ketoacidosis, n (%)	15 (4.6)	1 (2.0)	0.399
Inpatient stay-in general hospitalization rooms, median (IQR)^†^	14 (7-27)	8 (3-21)	0.004
Location of hospitalization, n (%)			
General hospitalization rooms, n (%)	66 (20.4)	23 (46.0)	< 0.001
ICU, n (%)	258 (79.6)	27 (54.0)	
ICU inpatient stay, median (IQR)*	12 (7-21)	12 (5-19)	0.673
Mortality, n (%)	102 (31.5)	8 (16.0)	0.025

* n = 196/324 and n = 29/50, respectively

^†^ n = 317/324 and n = 50/50, respectively

‡ Glycemic levels between 140-180 mg/dl

SOFA: Sequential Organ Failure Assessment; ARDS: acute respiratory distress syndrome; IVM: invasive mechanical ventilation; ICU: intensive care unit

### Diabetes versus stress hyperglycemia

We found that patients with diabetes presented a higher frequency of hypertension (63.9%; p = 0.001) and angiotensin II receptor blockers use (47.8%; p = 0.006) compared with the stress hyperglycemia group. There were no differences regarding age, heart disease, chronic kidney disease or angiotensin-converting enzyme inhibitors use.

With respect to inflammatory markers, NLR, D-dimer, and ultra-high sensitivity troponin-l values were higher in patients with stress hyperglycemia.

Inpatient global stay was significantly higher in those with stress hyperglycemia (p < 0.001), and there was no difference in intensive care unit stay time. Patients with stress hyperglycemia had higher chances of requiring invasive mechanical ventilation (OR = 2.85; 95% CI: 1.23-6.61; p = 0.001); likewise, the risk of dying was higher in this group (OR 2.15; 95% CI: 1.20-3.84; p = 0.009) (figure 3).

When adjusting for BMI, normal-weight or overweight in the stress hyperglycemia group, compared with patients with diabetes in the same BMI category, had a higher probability of dying. Obese patients with stress hyperglycemia did not have higher statistically significant chances of dying.

Related to age, having over 65 years was associated with a higher risk of death in both groups, being significantly higher in those older than 65 with stress hyperglycemia (OR = 8.80; 95% CI: 2.40-32.29; p = 0.001); in the 18-49 year- old group, this probability was three times higher (OR = 4.89; CI: 1.09-21.95; p = 0.001) for those who had stress hyperglycemia; and it was not significant in the 50-64 year-old group (OR = 2.37; 95% CI: 0.58-9.60; p = 0.228). Stress hyperglycemia increases the risk of death independent of the sex.

## Discussion

To our knowledge, this is the first study that compares the clinical outcomes of COVID-19 patients suffering from diabetes or stress hyperglycemia with those without these conditions in Latin America and the Caribbean.

The proportion of diabetes in the study was 27%. Previous studies have shown the prevalence of diabetes in patients hospitalized due to COVID-19 ranging between 5 and 20%, being higher as the severity of the disease increases ^21^.

The need for intensive care unit transfer in our population was 42%, a high number compared to what has been published for COVID-19 in general ^22^^,^^23^. Nonetheless, it can be because our institution is a regional reference center for high-complexity pathologies.

Diabetes and stress hyperglycemia have been associated with higher mortality. The finding of higher mortality in the diabetes group (adjusted OR = 3.16; 95% CI: 2.08-4.81) and the stress hyperglycemia group (adjusted OR = 8.12; 95% CI: 5.12-12.88) that developed COVID-19 is consistent with the reported literature in other population groups. A meta-analysis that included 83 studies performed in China, USA, France, Italy, Australia, and the United Kingdom with 78,874 patients admitted to inpatient treatment due to COVID-19 found that preexisting diabetes was related to approximately twice the risk of having severe or critical COVID-19 (n = 22 studies; random effects OR = 2.10; 95% CI: 1.71-2.57; I^2^= 41.5%) and with threefold the risk of inpatient mortality (n = 15 studies; random effects OR = 2.68; 95% CI: 2.09-3.44; I^2^= 46.7%) ^24^. Another meta-analysis that included 33 studies, conducted mainly in China, showed that diabetes in patients with COVID-19 was associated with an increase in twice the mortality and severity of COVID-19, compared to the without diabetes group (combined OR = 1.90; 95% CI: 1.3-2.64; p <0.01) ^25^.

The impact of diabetes on mortality increases if patients on top of it suffer from cardiovascular disease, chronic kidney disease, or hypertension. Another meta-analysis that comprised 35 studies conducted in China, France, Italy, Greece, and USA discovered that cardiovascular disease was strongly associated with both severity and mortality in COVID-19 patients (random effects OR = 4.02; 95% CI: 2.76-5.86; I^2^ = 53.08; and random effects OR = 6.34; 95% CI: 3.71-10.84; I^2^= 50.14), meanwhile, diabetes and hypertension were moderately associated with severity (diabetes: random effects OR = 2.35; 95% CI: 1.80-3.06; I^2^=34,78; hypertension: random effects OR = 2.98; 95% CI: 2.37-3.75; I^2^ = 49.89) and mortality (diabetes: random effects OR = 2.50; 95% CI: 1.74-3.59; hypertension: random effects OR = 2.88; 95% CI: 2.22-3.74; I^2^= 35.57) ^26^. Regarding chronic kidney disease, a meta-analysis of observational studies that included 13 studies adding up to a total of 18,822 patients found that the presence of diabetes in patients with chronic kidney disease with COVID-19 was correlated with a greater risk of mortality (RR = 1.41; 95% CI: 1.15-1.72; I^2^ = 70%) ^27^.

In our population, BMI was not a determinant for mortality, as are stress hyperglycemia and diabetes, independent risk factors for death. These results could be an information bias derived from the study design, the lack of standardization in the protocol to measure the height and weight of patients during the pandemic’s peak, and the small sample size in the group of patients with BMI recorded in charts. The result found in patients with obesity, without alterations in glucose, was not previously reported and could be explained by the small sample size.

When analyzing the impact of age on mortality, we found that the risk of dying was higher among older patients (> 65 years old) and that it increased considerably if the patient had a glucose alteration (diabetes or stress hyperglycemia). The impact of age on mortality in patients with COVID-19 was assessed in a meta-analysis that included 27 studies driven in 34 different geographical sites. This study reported an exponential relation between age and COVID-19 mortality, being very low in children and young adults younger than 25 years old (0.002% up to 10 years old and 0.01% until 25 years old) but raised progressively to 0.4% for those who are 55 years old, 1.4% up to 6 years old, 4.6% for 75 years old and 15% for those who are 85 years old ^28^.

We found a higher risk of death in patients aged between 18 and 49.9 years old compared to those aged 50-64, probably because the younger patients consulted later to health services (versus those older than 50), which could have impacted this group. Moreover, this could be explained by our sample size.

The presence of diabetes and stress hyperglycemia was associated with a higher need for ICU management and worse clinical outcomes (acute respiratory distress syndrome, invasive mechanical ventilation, vasopressor and inotrope support, *de novo* renal replacement therapy). A study done in Colombia evaluating associated factors with admission and mortality in intensive care unit in COVID-19 patients found severe pneumonia (OR = 9.86; 95% CI: 5.99-16.23), each point increase in the NEWS-2 score (OR = 1.09; 95% CI: 1.00-1.19), history of heart disease of ischemic origin (OR = 3.24; 95% CI: 1.16-9.00), and COPD (OR 2.07; 95% CI: 1.09-3.90) among the factors related to intensive care unit admission; while for mortality: age younger than 65 years (OR = 3.08; 95% CI: 1.66-5.71), acute kidney injury (OR = 6.96; 95% CI: 4.41-11.78), intensive care unit admission (OR = 6.31; 95% CI: 3.63 - 10.95) and for every point increase in the Charlson comorbidity index (OR 1.16; 95% CI: 1.00-1.35), but only 20.5% of the cases had a history of diabetes ^29^. A meta-analysis that included 78 studies of critically ill patients, with 21,510 patients treated in intensive care unit, showed that the mortality rate in patients with mechanical ventilation was as high as 47.9% (95% CI=41.6 - 54.2; I^2^ = 96.9%) and renal replacement therapy was 58.7% (95% CI: 50.0-67.2; I^2^= 83.1%) ^30^. Another study performed in New Jersey showed that 79.5% of intubated patients had diabetes ^31^.

When comparing the outcomes between those with hyperglycemia (diabetes versus stress hyperglycemia), we found that the presence of stress hyperglycemia is linked to a higher risk of complications and death when compared to the presence of diabetes (figure 2). A probable explanation could be that hyperglycemia is a stress and inflammatory marker potentially contributing to adverse metabolic responses to infection ^32^. It is consistent with an observational study performed in New York with 133 patients describing that patients with stress hyperglycemia have an adjusted hazard ratio (HR) higher for 14-day mortality (HR = 7.51; 95% CI: 1.70-33.24) and 60 days (HR = 6.97; 95% CI: 1.86-26.13) when compared to the group without diabetes. Similarly, there were higher levels of C-reactive protein, procalcitonin, and lactate ^33^.

A study conducted in France showed that at least a quarter of COVID-19 hospitalized patients had diabetes, and additionally, it was associated with a higher risk of intensive care unit admission but not with mortality ^34^. Our study found that most cases corresponded to stress hyperglycemia instead of diabetes and that this group required intensive care unit management to a greater extent. Furthermore, there was a higher mortality in the diabetes group. This association was described in England’s National Cohort study (adjusted HR = 1.23; 95% CI: 1.14-1.32) since 26.4% of deceased patients had diabetes ^35^.

Patients with stress hyperglycemia and diabetes received steroids more frequently than those without diabetes, which could have influenced the results. However, the type and dose of these are unknown, and a specific analysis of their effects on adverse outcomes cannot be made considering the studies that suggest lower mortality with its use ^36^^,^^37^.

The mechanism by which the population with glucose abnormalities has worse outcomes is poorly understood. Nevertheless, historically, hyperglycemia alters the immune system response to infection (compromises chemotaxis, phagocytosis, innate cellular immunity), increases the release of pro-inflammatory chemokines, generates abnormalities in the coagulation system, increases oxidative stress, and at a pulmonary level, induces a prolonged inflammatory response, bronchial hyperreactivity and the development of fibrosis in the airway ^38^, all of which potentially explains the unfavorable outcomes seen in patients with diabetes and viral infections in previous pandemics (i.e., Middle East respiratory syndrome coronavirus, MERS, or AH1N1) ^10^^,^^24^^,^^39^^-^^41^.

Stress hyperglycemia presents a higher prevalence of rise in acute phase reactants, suggesting that this phenomenon is derived from immune system dysregulation. These observations are related at a molecular level with various mechanisms, including reduction in neutrophil degranulation, expression of cytokines, phagocytosis, and cellular toxicity ^40^.

Likewise, it worsens the patient’s inflammatory state and oxidative stress, generating an increased hyperglycemia that augments cellular glucotoxicity. Simultaneously, insulin resistance increases circulating free fatty acids causing lipotoxicity, which constitutes, together with inflammation and glucotoxicity, the most important characteristics of acute illness related to hyperglycemia. Additionally, insulin resistance and secondary hyperinsulinemia can promote endothelial dysfunction and alterations in the fibrinolytic system ^42^, meaning all previous elements add up for worse clinical outcomes.

In our population, those patients with diabetes and stress hyperglycemia had significantly higher levels of NLR, serum concentrations of C-reactive protein, D-dimer, and ultra-high sensitivity troponin-l compared to the group without diabetes. This finding suggests a greater inflammatory response that was apparently higher in stress hyperglycemia patients since inflammatory response markers were higher when compared to the patients with diabetes.

Our study has certain limitations, and our descriptions must be interpreted in the context of its design. First, our institution is a reference center for the management of Colombian southwest patients, the reason for which there could be a selection bias within our population. Second, clinical data from every patient was obtained directly from clinical records and secondary databases (clinical and microbiology laboratories). Ergo, there can be an information bias from missing relevant patient data-as it happened with BMI and HbA1c, which was strikingly lower than what was reported in other local studies, not knowing if this could impact the outcomes evaluated-and their present comorbidities. Due to this, there is no detailed specification with respect to the diabetes type of the included patients. However, most of them probably correspond to type 2 diabetes, considering the local prevalence when compared to type 1 diabetes in the country ^43^. Third, the only clinical tests considered for the study were those taken at hospital admission, but those laboratory parameters were not followed up during the inpatient stay.

The main limitation of this study is that when designing the methodology, including the variables and planning the statistical analysis, we considered the conditions that were relevant at that pandemic time, in the midst of global ignorance of the disease course of SARS-CoV-2 infection, may be left out of the analysis confounding factors of individual or pathological character that could influence the outcomes in a positive or negative way.

One of the strengths from the study was the adjustment for common confounding factors in the population, some already suggested as risk factors for adverse outcomes in COVID-19, such as hypertension, cardiovascular disease and chronic kidney disease. Data adjustment to age also resulted in a strong point of the study due to the known and reported relation between higher age and worse outcomes in COVID-19. Even though the retrospective aspect of the study impedes us from excluding every potential confounding factor, the strength of association found between diabetes and stress hyperglycemia and adverse outcomes that prevail after adjusting for co-variables supports the hypothesis that alterations in glucose metabolism within the hospital, such as diabetes and especially stress hyperglycemia, constitute risk factors for the development of severe COVID-19 and unwanted clinical outcomes. The number of patients included is a strength because of the lack of data reported in Latin-American populations of this kind.

Another limitation of the study was the unanalyzed coexistence of infections and their possible impact on outcomes. Concerning other drugs received by patients, we explored the effect of taking angiotensin-converting enzyme inhibitors or angiotensin II receptor blockers (because the initial literature during the pandemic reported some data that suggested worse outcomes in patients who received them) without finding significant differences between those taking it and those who did not.

The previous use of other drugs, such as immunosuppressants, was an exclusion criterion to avoid these as confounding factors.

Despite our limitations, our study contributes to the knowledge of the behavior of COVID-19 patients with diabetes or stress hyperglycemia in Colombia and Latin America, aiming to establish public health strategies at a clinical level to favor better clinical outcomes in this population.

Diabetes and stress hyperglycemia are associated with unfavorable clinical outcomes and higher mortality in patients with COVID-19. Among the alterations, the presence of stress hyperglycemia grants a significantly higher risk. Hence, the importance of considering this group of patients as high risk at the moment of COVID-19 diagnosis to initiate early therapeutical measures and mitigate adverse outcomes.
